# Facilitations in the Clinical Diagnosis of Human Scabies through the Use of Ultraviolet Light (UV-Scab Scanning): A Case-Series Study

**DOI:** 10.3390/tropicalmed7120422

**Published:** 2022-12-08

**Authors:** Gaetano Scanni

**Affiliations:** 1 Dermatology Unit, Department of Prevention ASL, 70123 Bari, Italy; g.scanni@entodermoscopy.net; 2 Osservatorio Per lo Studio e la Prevenzione Delle Parassitosi ed Infezioni Nelle Collettività (OPIC), Via Lungomare Starita n.6, 70132 Bari, Italy

**Keywords:** scabies, ultraviolet-A light, Mite-Gallery Unit, full frame view, dermoscopy, diagnosis, neglected disease

## Abstract

Background: To confirm the suspicion of scabies, dermatologists have one pathognomonic sign, “the tunnel” through which *Sarcoptes scabiei* digs into the epidermis. Light microscopy is considered the most reliable procedure, but it is time-consuming and operator-dependent. Recently, dermoscopy has greatly improved the chances of recognizing mite in situ, but it is still linked to the examiner’s experience and to the magnification capability of the device used. Methods: This article, based on a case-series study, describes a novel diagnostic path, which uses an ultraviolet LED source at 365 nm and a digital camera for the evaluation of lesions that raise the suspicion of scabies. Results: The gallery emits a naked-eye-visible wavy bluish-white linear luminescence, better than that of any standard lighting. UVA light is also able to identify *Sarcoptes scabiei* as a white or green point-shaped area. This sign can only be appreciated by enlarging its picture to full frame on a common PC monitor. Conclusions: Ultraviolet light (365 nm) seems to offer help in the diagnosis of scabies because it saves time compared with light microscopy and because it does not require contact with the patient’s skin, as in dermoscopy. Although examiner experience remains an important factor, it is easily compensated by procedural simplicity, the cost of the devices and, especially, by the clarity of the results, even in non-specific lesions.

## 1. Introduction

In 2020, the International Alliance for the Control of Scabies (IACS) established that the definitive diagnosis of scabies is possible only when *Sarcoptes scabiei* and/or its products (ova and scybala) are demonstrable on the patient’s skin through microscopic or dermatoscopic procedures (A 1-2-3) [1]. In all other conditions, the reliability of the diagnosis decreases according to the symptoms and epidemiology of the patient (B 1-2-3; C 1-2; H 1-2) (Table 1).

This scenario demonstrates the difficulty in recognizing the infestation because it is strongly conditioned by the demonstration of the only pathognomonic sign represented by a superficial tiny whitish curvilinear line in target sites, such as the interdigital spaces, wrists, elbows, armpits, mammary region and genitals [2].

Recently (2019) dermatoscopy showed that a “burrow” is not a single entity but a structure composed of three different parts for morphology and function generally known as the Mite-Gallery Unit (MGU) [3]. The tunnel segment in which the mite lives is called the “head”; it generates a triangular image that resembles a hang-glider named “delta wing sign” in wet dermoscopy (liquid film + glass plate) [4].

**Figure 1:**
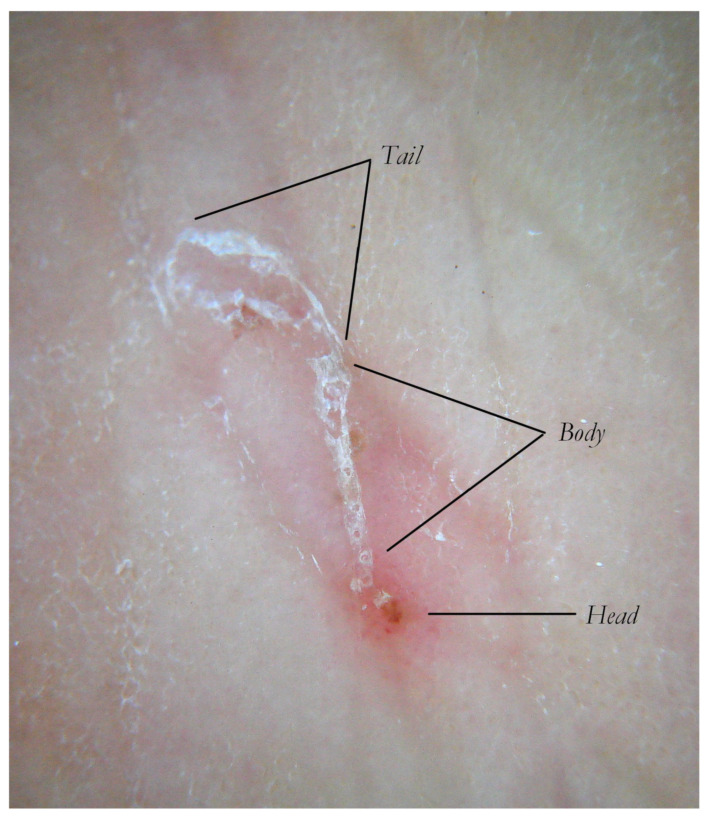
The Mite-Gallery Unit in non-polarized dry dermoscopy (d-DS). MGU can be divided into three parts. The Head hosts the mite, the Body contains parasite eggs and feces and the Tail, which has not roof but features keratinic collarettes that are visible only in d-DS. An erythema is present in the background around and immediately behind the mite.

This section is followed by the main part of the tunnel, called the “body”, whose roof features small, scattered holes that produce the “jet-contrail” sign. Eggs and numerous tiny whitish spheres corresponding to the scybala (fecal pellets) can be found in this location. Finally, this is followed by the third segment, called the “tail”, which lacks a roof and is visible only by dry dermatoscopy (no liquid and no glass plate) as two parallel keratin edges, which progressively diverge [5] according to the lesion’s duration and the local host’s reaction (Figure 1).

**Figure 2:**
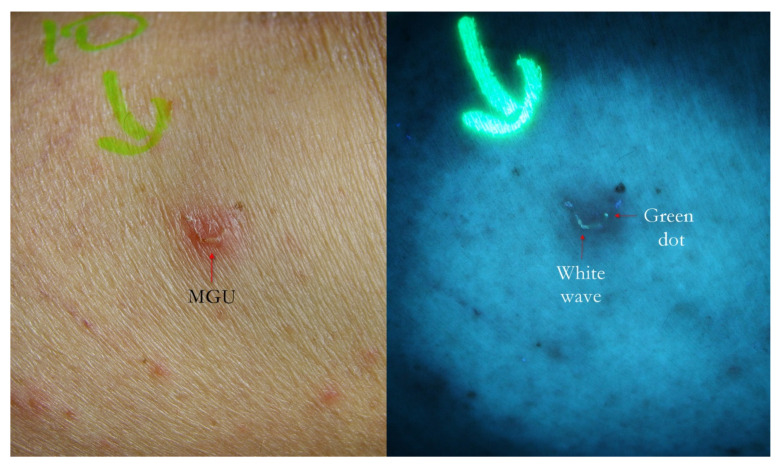
Flash light vs. UVA. A MGU was already recognizable to naked eye (**left**) as an apparent single structure. However, UVA light was able to distinguish body-MGU (white wave) from head-MGU (green-dot) where *Sarcoptes* body was physically located (**right**). The red phlogosis halo around burrow returned a darker light than the rest of background. A yellow highlighter was used.

**Figure 3:**
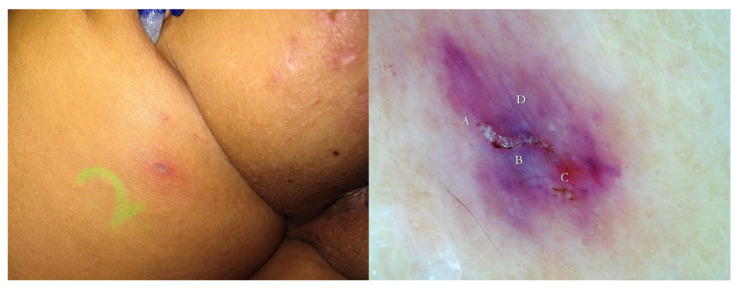
Post-therapy non-specific lesion under buttock after two 5% Permethrin applications 7 days apart (**left**). Under wet dermoscopy, inside erosion, there was an unexpected MGU with the delta sign of *Sarcoptes* (A) and a gallery with fecal pellets (B) that faded in a fuzzy tail (C). All around, there was a purplish area (D) corresponding to superficial hematoma caused by patient scratching (**right**). A yellow highlighter was used.

**Figure 4:**
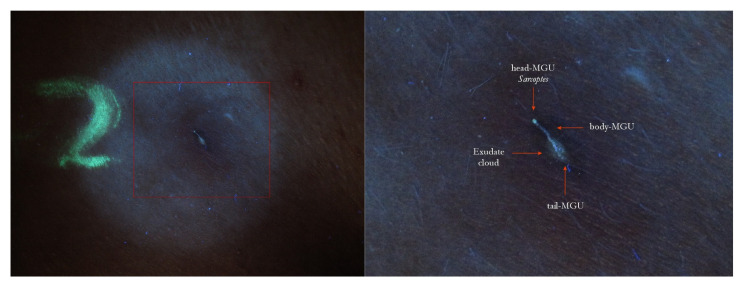
The same lesion under UVA light showed a linear white luminescence (**left**, red box). In full-frame vision (**right**), it was possible to distinguish a Mite-Gallery Unit whose head part was occupied by *Sarcoptes*, which featured a green hue (green dot). Between body and tail MGU, there was a luminescent cloud caused by exudate and phlogosis around burrow (rocket sign). A yellow highlighter was used.

The knowledge of the ultra-structure of the “burrow” is useful for interpreting the images described in this study (Figure 2, Figure 3 and Figure 4).

Although the diagnostic key of scabies is well defined by a specific sign, it is not always possible to locate both because of the low number of burrows [6] and because the patient’s scratching makes them very difficult to observe with the naked eye.

Throughout the history of medicine, many attempts have been made to solve this problem, starting from the elementary burrow ink test up to the most recent approach, confocal microscopy. Even today, however, the gold standard is the light-microscopy examination [7], which requires many minutes of preparation and reading and, in particular, a fair amount of luck in order to locate a whole mite rather than single parts of it or its eggs or feces, which are difficult to recognize if they are observed separately from the parasite. For this reason, any dermatologist would desire a simple and fast test, with objective diagnostic reliability that is not dependent on operator experience.

This article describes a new solution for MGU identification, proposed as a support for traditional methods, which is able to highlight both tunnel and mite better than any natural or artificial light by using ultraviolet light (UVA). The discovery of this UVA property occurred by chance during the author’s investigations, which were not specifically aimed at scabies diagnosis. Events of this type are instances of “serendipity”, which are not uncommon in the history of medicine [8]. At the time of writing, no previous publications regarding in vivo and in situ diagnosis of scabies by UVA light have been found using the main scientific-web-search engines.

## 2. Materials and Methods

This study was based on a sample of patients suffering from scabies in the interval between May 2021 and May 2022 (12 months). The age range was between 1 and 92 years for a total of 53 patients referred to the Observatory for the Prevention and Study of Parasitosis and Infections in the Communities (OPIC) operating in the Department of Prevention of the Local Health Authority of Bari (ASL Bari). Some of the patients (about 1/3) came from elderly-long-term-care facilities. The “natural” fluorescence of scabies described in this article should not be confused with the artificial one induced by tetracyclines topically applied to highlight burrows [9], nor with that of microscope samples treated with fluorochromes [10].

In vivo and in situ ultraviolet-light positivity concerns both the tunnel and the body of *Sarcoptes scabiei*. The former is already appreciable to the naked eye, while the latter requires an enlargement that is obtained by observing each photograph of suspicious lesions in full frame view (FFV). For this purpose, two 12-megapixel digital cameras (Canon Ixus 110 IS/Olympus XZ-2) were employed, along with FFV managed on a 32-inch PC monitor by ACDsee Photo Studio Ultimate-2021 editor program. Normally, the FFV mode reveals greater detail because the image expands to all native resolutions offered by a given sensor size. Beyond this physical limit, clarity is impaired by digital background noise.

A torch supplied with latest LED generation at 365 nm/10W output was used as ultraviolet-light source (Alonefire) [UVA test is in Supplementary Materials section]. The lamp was positioned a few centimeters in front of or alongside the examined area while the photographic UVA records took place in dim light without flash with an underexposure between -1 and -2 EV and a variable sensitivity between 100 and 800 ISO. With this setting, it was possible to shoot freehand with a shut speed between 1/30 and 1/80s and to obtain satisfactory results, provided that the patient and the observer remained still for a while. By contrast, the ordinary clinical photographs were obtained with flash-on and all camera parameters were set in automatic mode.

When 365-nanometer UVA light is directed onto intact skin, a low-intensity uniform bluish color returns, representing the “neutral” background to which each sign has to be compared. The positivity of any skin lesion is described by the word “luminescence” because it is brighter and of a different color from healthy skin. The prevalent colors observed in scabies were bluish-white (also referred to as white for simplicity) and green in varying shades. To facilitate the comparison between ordinary light and UVA pictures, the skin was previously marked with a yellow highlighter that produced a very intense luminescence if inside the UVA image.

Dermatoscopy was performed by a Delta 20 (Heine Optotechnik GmbH & Co, Bavaria, Germany) dermatoscope in wet (glass plate + liquid film) or dry (no plate, no liquid) modality was used.

## 3. Results

In ordinary lighting, a scabies gallery can be recognized by the naked eye (if it remains intact) because the mite creates a very thin stratum corneum under which air can enter. In fact, when the air is pushed out during wet contact dermoscopy, the tunnel becomes invisible [3]. UVA skin analysis has proven to be free from this limiting event because it identifies the luminescence of dislodged keratin in any situation.

**Figure 5:**
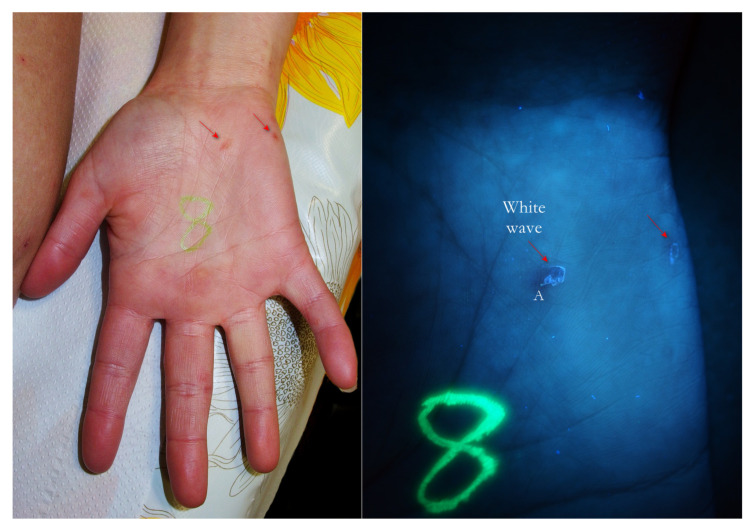
Flash light vs. UVA. Two MGUs appeared as pink fuzzy streaks (**left**, red arrows). Under UVA they became clear linear structures (white waves), as in the pathognomonic sign of scabies. At one extremity, a little white dot was recognizable (A). A yellow highlighter was used.

**Figure 7:**
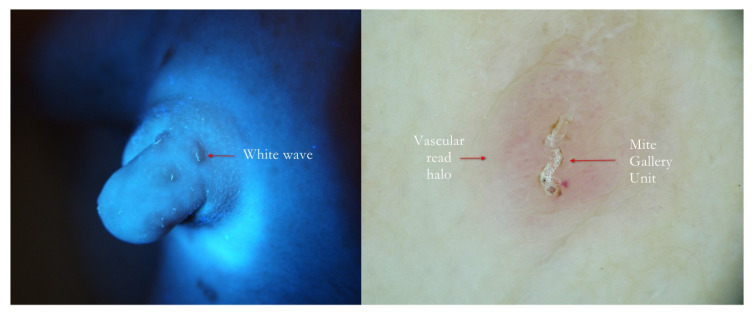
Scabies penile papules. The at-a-glance bright linear UVA track (**left**, red arrow) was confirmed to be a typical Mite-Gallery Unit surrounded by a pink vascular infiltrate under wet dermoscopy (**right**).

**Figure 8:**
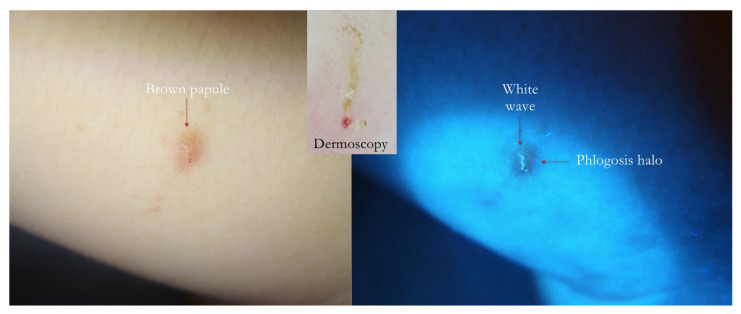
Flash light vs. UVA. Papular scabies. A generic brown infiltrative lesion on inner side of thigh (**left**) produced a wavy linear luminescence under UVA (**right**). In the upward inset, dermatoscopy showed that the white wave was a MGU housed in the papule darker than the background.

**Figure 9:**
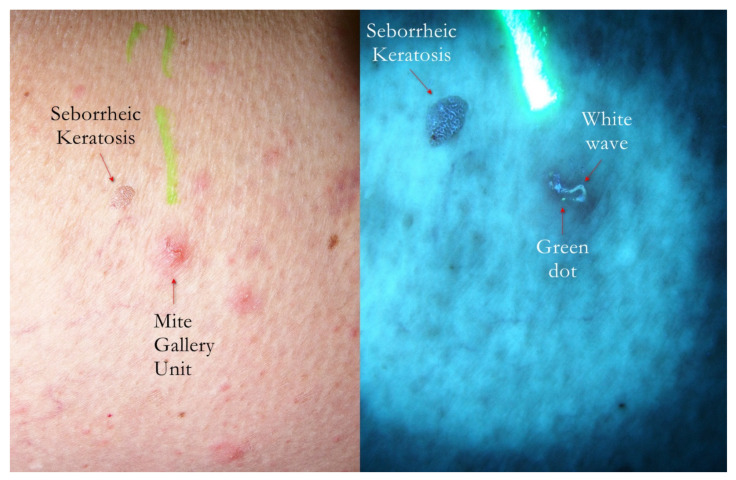
Flash light vs. UVA. A little erythematous area on which a fuzzy linear lesion appeared to the naked eye (**left**). UVA light highlighted the undulated line that corresponded to a MGU whose one extremity ended with a green dot where *Sarcoptes* was located. A yellow highlighter was used.

**Figure 10:**
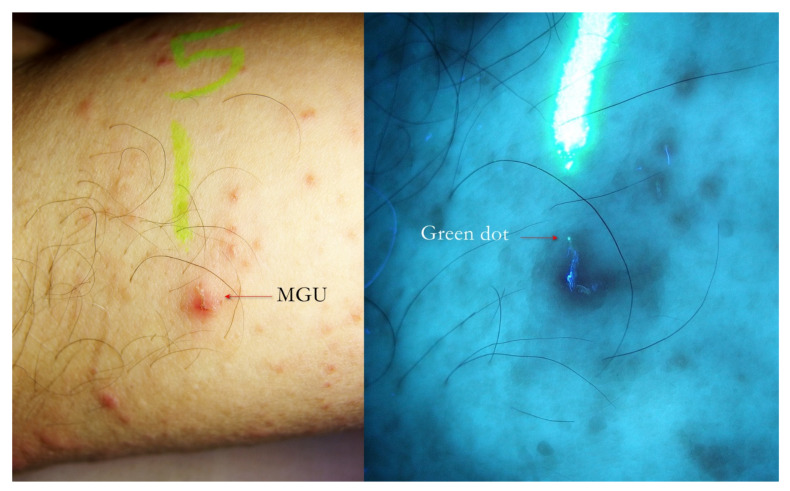
Flash light vs. UVA. A MGU was distinguishable to naked eye (**left**). UVA light drew attention to the green dot where *Sarcoptes* body was located. The gallery behind had a bluish hue partially caused by a fabric thread glued on MGU. A yellow highlighter was used.

**Figure 11:**
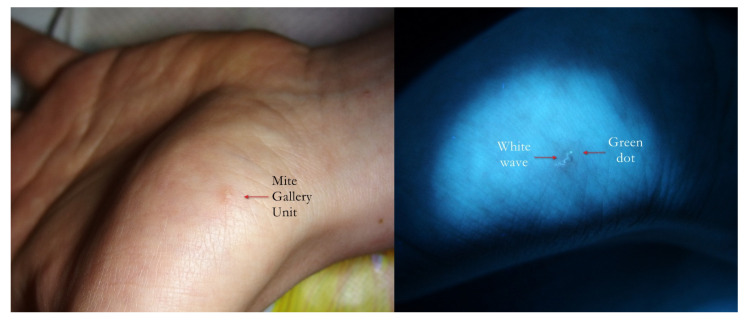
Flash light vs. UVA. On thenar eminence, a very suggestive linear lesion (**left**) that under UVA (**right**) clearly became a wavy gallery (white wave) ending with a green dot where the mite was physically located.

**Figure 12:**
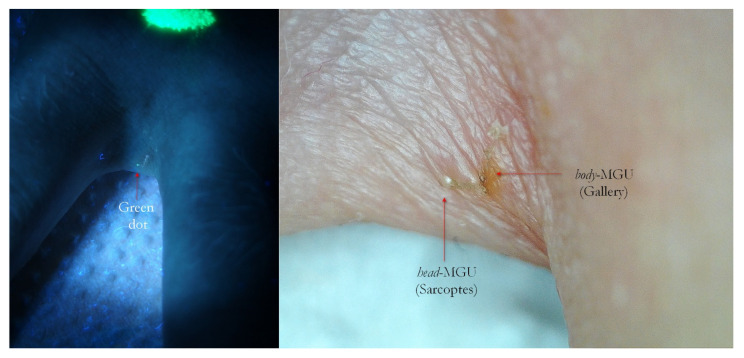
Scabies of interdigital fold of hand. Interdigital MGU under UVA exhibited a green dot (**left**, red arrow). A bright blue luminescent fabric thread was nearby. Dry dermoscopy of same MGU confirmed *Sarcoptes* and its gallery (**right**, red arrows), but site anatomy made it difficult to obtain all-in-focus image. A yellow highlighter was used.

**Figure 13:**
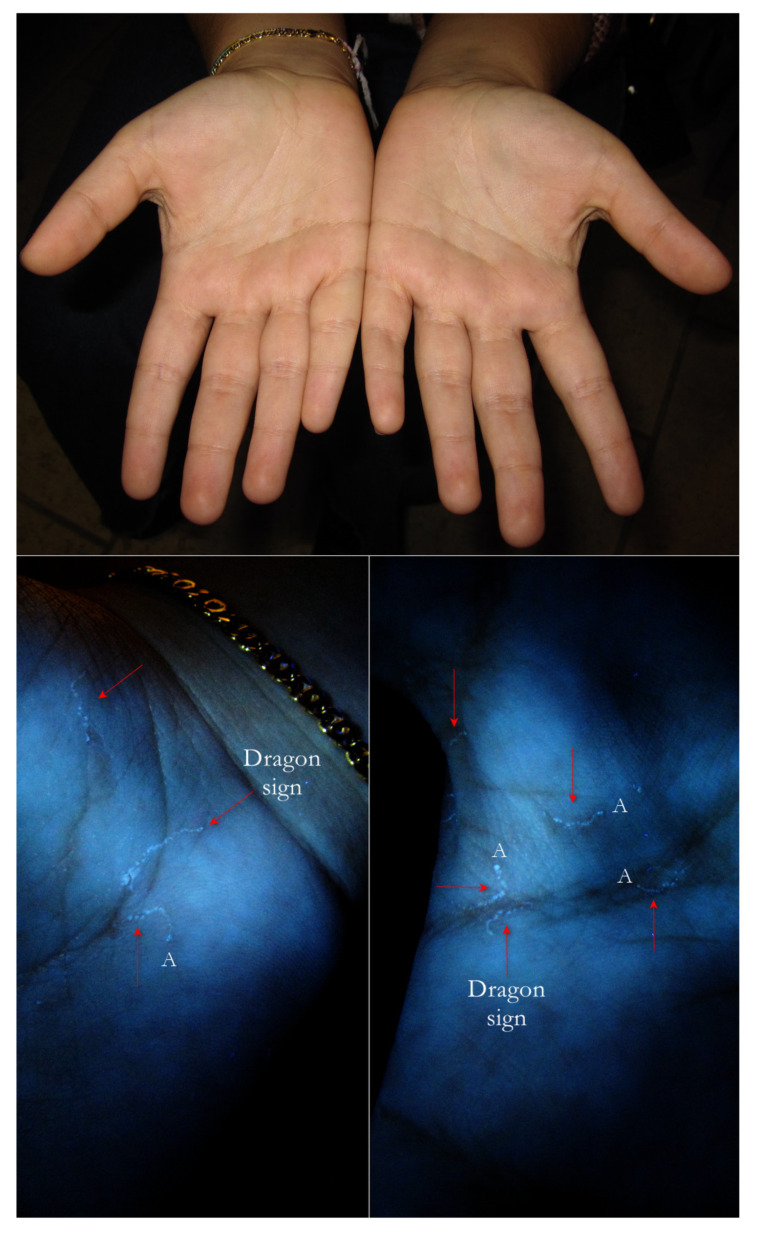
Flash light vs. UVA. Two palms under flash light did not show any problems. However, under UVA, some Mite-Gallery Units (red arrows) were immediately visible as segmented lines (dragon sign). At one extreme, some of them exhibited a little white dot (A) where the *Sarcoptes* body was located. The lines drawn were finely segmented because the gallery roof was drilled.

**Figure 14:**
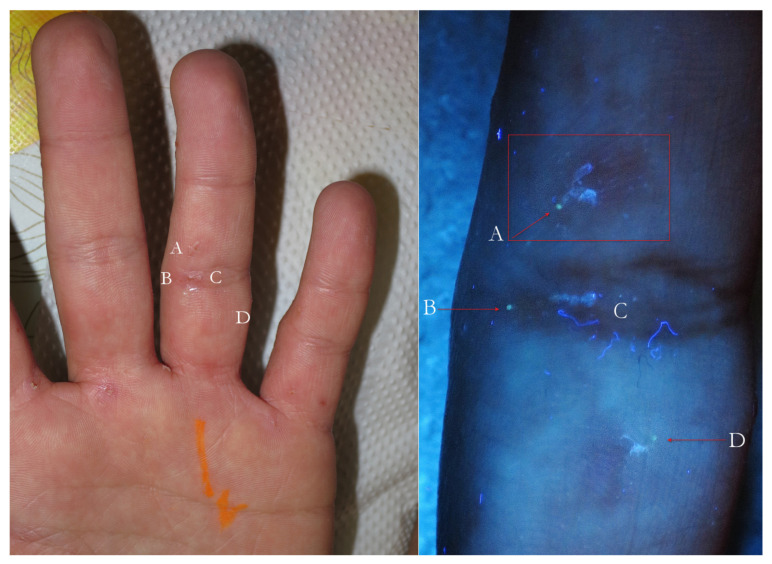
Flash light vs. UVA. On the left, some very suggestive signs of MGUs that in full-frame view on the right corresponded to two green dots beside galleries (A–D), one isolated green dot (B) and a white line alone (C). B and C probably belonged to the same Mite-Gallery Unit. Sharp blue tortuous lines are fabric threads on background. A red highlighter was used.

**Figure 15:**
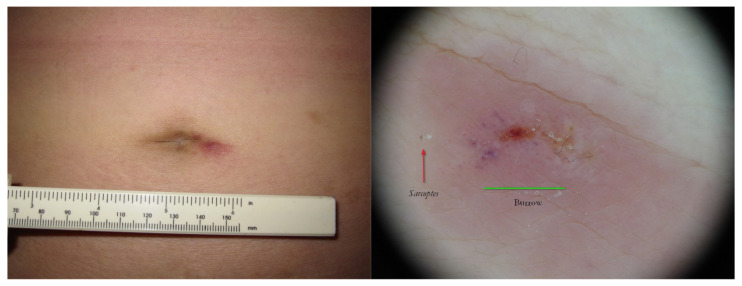
Surrepticius scabies. A non-specific lesion on navel in an asymptomatic patient (**left**). Wet dermoscopy of the same lesion (**right**) showed a *Sarcoptes* as a brownish triangle (red arrow) and a burrow was no longer distinguishable because it was modified by host phlogosis-exuding reaction (green segment).

**Figure 16:**
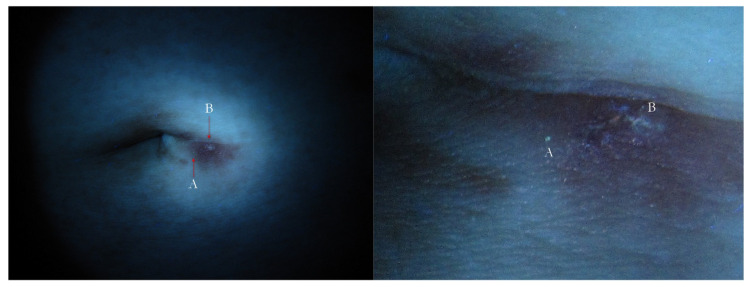
Surreptitious scabies. The same patient under UVA. The navel lesion shows too little signs (**left**) that in full-frame view can be identified as a green dot (A) and a fuzzy white line (B), corresponding to *Sarcoptes* body and gallery remnants, respectively (**right**).

The results obtained in this study by UVA light can be summarized in six morphological sets:

1. The gallery corresponding to the “body” of the Mite-Gallery Unit under UVA produced a bluish-white luminescence easily distinguishable from the background light. This sign was called “white wave” due to the curvilinear trace (Figure 2, Figure 5, Figure 6, Figure 7, Figure 8, Figure 9, Figure 10, Figure 11 and Figure 12).

2. In some cases, at one end of the tunnel, a point-like area was appreciable, in which the brightness was more compact. This sign, corresponding to the place where the *Sarcoptes* resides, was called “white dot” (Figure 5 and Figure 13).

3. In the palm-plantar areas, the gallery appeared as a bluish-white luminescent discontinuous line due to the numerous sweat-gland openings. For the wavy and segmented pattern, this sign was metaphorically defined as “dragon sign” due to its resemblance to the dragons performed in Chinese festivals (Figure 13).

4. In some cases, it was possible to observe a bluish-white MGU that showed a small greenish luminescent point at one extreme slightly apart from remaining line (Figure 2, Figure 9, Figure 10, Figure 11 and Figure 12). This sign, defined as a “green dot”, corresponded to the position of the *Sarcoptes*. Sometimes, the green dot appeared as a single element, i.e., without a tunnel behind it (Figure 14, Figure 15 and Figure 16). This event can partly be explained by the damage caused by the host or by the immature forms of *Sarcoptes* (larvae and nymphs), which do not dig tunnels but remain still in a small hole until they become adults (moulting niches) [11].

5. Some MGUs may occur near excoriated or exuding areas. In these cases, the tunnel is easily recognizable under UVA because around the linear luminescence, a nebulous luminescence is added due to the exudation of liquids. In metaphorical language, this aspect was defined as the “rocket sign” because the light of the exudate recalls the exhaust gas of a launch vector (Figure 3 and Figure 4). When the inflammation manifested as erythema, the emitted luminescence was darker than the background one.

6. In recent papular lesions, the MGU was clearly recognizable as a wavy luminescent white line inside them (Figure 7 and Figure 8). When the inflammation manifested as a papule, it appeared as a blurry area darker than the background light.

## 4. Discussion

The primary goals of this study have been to demonstrate that UVA light is able to detect pathognomonic signs of scabies better than normal light and to define the real nature of linear and point-like luminescences. For these reasons, the skin pictures taken by flash were compared side by side to UVA pictures with a mark outlined with a common yellow highlighter as a reference point. Next, a dermoscopic analysis of a suspicious lesion concluded the demonstrative logic tree.

It was immediately evident that the naked-eye recognition of burrows was really much more efficient under UVA than under any natural/artificial light commonly used for patient clinical examination. In fact, when galleries do not receive ordinary light with a proper inclination, they can go unnoticed, especially if they are short, if they are masked by concomitant lesions, or if their roofs have been destroyed by the host. With UVA light, tunnel identification is not affected by these interferences, demonstrating a wavy luminescent line (white wave).

Using dermoscopy, in all cases, it was possible to demonstrate the correspondence between the structures observed in the UVA and the dermatoscopic ones described in the literature, that is, the wavy linear luminescence corresponded to the galleries (Figure 2, Figure 5, Figure 6, Figure 7, Figure 8, Figure 9 and Figure 11), while the point-like areas at the ends of the tracks corresponded to the mite’s body (Figure 2, Figure 4, Figure 5, Figure 9, Figure 10, Figure 11, Figure 12, Figure 13, Figure 14 and Figure 16

Given the efficiency of light-skin UVA scanning, examination with high-power Wood’s lamps could become the standard procedure for all subjects complaining of unexplained itching. Currently, new 365-nanometer LEDs are available with high outputs (10 W or higher). These can be used to highlight mite tunnels sufficiently to become visible to the naked eye and photographable by ordinary digital cameras.

**Figure 17:**
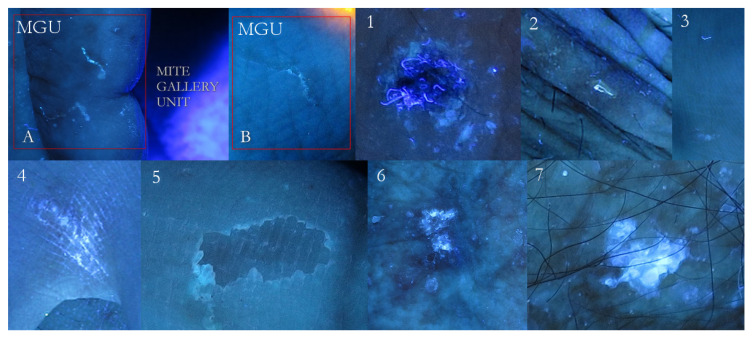
False-positive examples. A mosaic table of possible positive signs compared to Mite-Gallery Units (**A** + **B**). Fabric threads captured by an erosion (**1**). Single thread shinier then any MGU (**2**). A thread above, a remnant of burrow down (**3**). Bright luminescent exogenous substance contaminating interdigital hand folds (**4**). Very superficial erosion as a faint luminescent scalloped collarette (**5**). An evident white luminescence of an epidermal keratosis (**6**). A very bright white lamellar luminescence of an actinic keratosis (**7**). These signs can be found separately or close to specific scabies lesions.

Another important aspect found during the UVA examination concerns the accidental luminescences (false positivity) due to exogenous contamination or to other concomitant previous dermatological pathologies (Figure 17). Some local situations that require a differential diagnosis are already known. The most common is caused by textile debris detaching from clothing. In general, these structures have a very bright and compact linear or punctiform bluish luminescence. They do not belong to the epidermis but rest on it; therefore, they are easily movable. Furthermore, cosmetic residues are very common and distinguishable because they copy the dermatoglyph network or show irregular shapes incompatible with that of a MGU.

On the other hand, it is more challenging to distinguish hyperkeratotic structures (Figure 17), which can be primary or secondary to the inflammation induced by *Sarcoptes*. Keratoses appear intensely bright with a bluish-white color, in which overlapping polygonal compact laminae can be recognized. When older MGUs are no longer active, the tunnel boundaries move away, producing areas with wavy keratin borders (ghost-MGU) [3].

In order to avoid evaluation mistakes, it is always advisable to compare the UVA results with those in natural/flash light, while to remove the background noise produced by the textile fibers, it is sufficient to clean the area with a common antiseptic liquid before the examination.

An interesting opportunity for the use of UVA light in scabies diagnosis is to clarify the real nature of non-specific lesions that can occur ab initio or post-therapy. In these clinical conditions, representing a true challenge for every dermatologist, the documentation of a white wavy luminescence (white wave) or of a fluorescent spot isolated or tunnel-associated (green dot/white dot) erases doubt. Depending on the extent of the patient’s anamnesis, it is possible to diagnose “surreptitious” scabies (Figure 15 and Figure 16) in the former case [12] and, in the latter (Figure 3 and Figure 4), inadequate topical applications [13] or, alternatively, drug resistance [14].

Moreover, the usefulness of MGU study via UVA light consists in supporting traditional diagnostic procedures (scraping + light microscope or digital dermatoscopy) guiding the dermatologist to choose an area actually inhabited by the mite, avoiding the false negatives caused by non-parasitic skin sampling.

Finally, this method is highly advantageous for the exploration of the ano-genital regions because it does not require any contact with the patient’s skin (Figure 3, Figure 4 and Figure 7), as well as for that of the interdigital folds, where the full contact of the dermatoscope’s glass-plate is very difficult to establish and the quality of the image can be low (Figure 12).

## 5. Conclusions

A discovery supported by serendipity seems to offer a new source of support for the diagnosis of the viability of scabies infestations through in vivo and in situ identification of burrows and mite bodies when illuminated by 365 nm of UVA light (UV-scab scanning).

This procedure is able to identify a tunnel visible to the naked eye thanks to its bluish-white luminescence much more efficiently than any other visible-light sources. The body of *Sarcoptes scabiei* also emits a dot-like luminescence (white or green), although this is visible only when magnifying a common digital photograph at full size on any monitor.

To a greater extent than in full clinical conditions, this procedure promises the best diagnostic results in paucisymptomatic forms characterized by ab initio ambiguous manifestations or in monitoring unclear lesions after treatment that create significant indecision regarding therapeutic efficacy and patient contagiousness.

If future studies confirm the convenience of this procedure, it will represent an important diagnostic development over the inexpensive instruments already present in any dermatologist’s office and a limited time required for the full-frame view of scabies images on PC monitors or other displays.

Based on these premises, the classic pathognomonic sign of scabies, “the burrow”, is more easily demonstrable in vivo and in situ thanks to fluorescence of *Sarcoptes scabiei* and its gallery.

The future availability of a device able to integrate all the steps of this novel procedure in a single action would offer a quick pre-dermoscopic/microscopic diagnosis upon the first examination of patient.

This represents a significant breakthrough in the history of scabies.

## Figures and Tables

**Figure 6:**
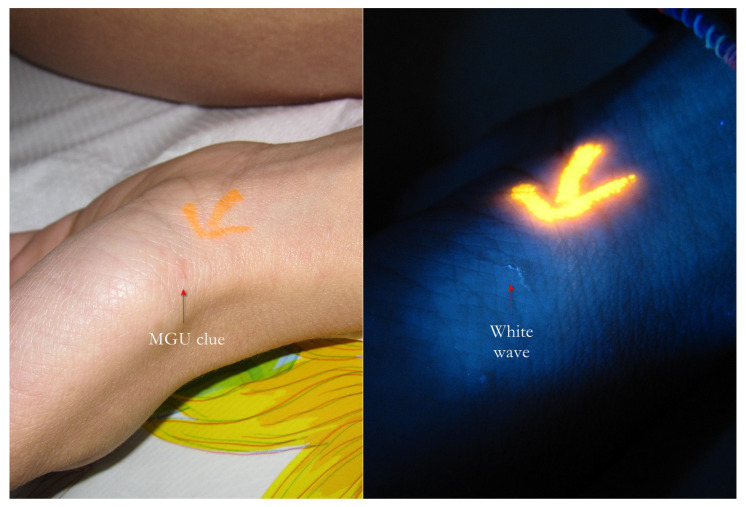
Flash light vs. UVA. The burrow was a clue (**left**, red arrow) that under UVA light, a very clear linear structure (white wave) formed, suggesting a typical Mite-Gallery Unit (**right**). A yellow highlighter was used.

**Table 1:** Diagnostic criteria established in 2020 by IACS (International Alliance for the Control of Scabies).

A. Confirmed Scabies
At least one of:
A1. Mites, eggs, faeces on light microscopy of skin samples
A2. Mites, eggs, faeces visualized on an individual using a high-powered imaging device
A3. Mites visualized on individual using dermoscopy

B. Clinical Scabies
At least one of:
B1. Scabies burrows
B2. Typical lesion affecting male genitalia
B3. Typical lesions in a typical distribution and two history features

C. Suspected Scabies
One of:
C1. Typical lesions in a typical distribution and one history feature
C2. Atypical lesions or atypical distribution and two history features

History Features
H1. Itch
H2. Positive contact history

## Data Availability

Not Applicable.

## Supplementary Materials

The following supporting information can be downloaded at: https://www.mdpi.com/article/10.3390/tropicalmed7120422/s1, File S1: UVA TEST+RULER.
