# Supplementary material for: Facilitations in the Clinical Diagnosis of Human Scabies through the Use of Ultraviolet Light (UV-Scab Scanning): A Case-Series Study
Source: Trop Med Infect Dis. 2022 Dec 8;7(12):422. doi: 10.3390/tropicalmed7120422 (PMC9785351; doi:10.3390/tropicalmed7120422)
Supplement: Supplementary file 1 [file tropicalmed-07-00422-s001.zip › tropicalmed-1984034-supplementary.pdf]

Tropical Medicine and Infectious Disease

MDPI

Supplementary Materials: UVA TEST+RULER

Gaetano Scanni [1,2]

[1] Dermatology Unit, Department of Prevention ASL, 70123 Bari, Italy; g.scanni@entodermoscopy.net
[2] Osservatorio Per lo Studio e la Prevenzione Delle Parassitosi ed Infezioni Nelle Collettività (OPIC), Via Lungomare Starita n.6, 70132 Bari, Italy

A new generation of high-efficiency UVA LEDs is able to generate a more intense and coherent beam of light capable of improving the results of a skin examination with the traditional Wood's light.

A 365nm UVA (10W output) torch was used in this article. The validity of the light source was previously assessed with an easy test by illuminating in the dark room a 50 euro banknote on which fluorescent symbols, invisible under natural light, are printed (Figure S1).

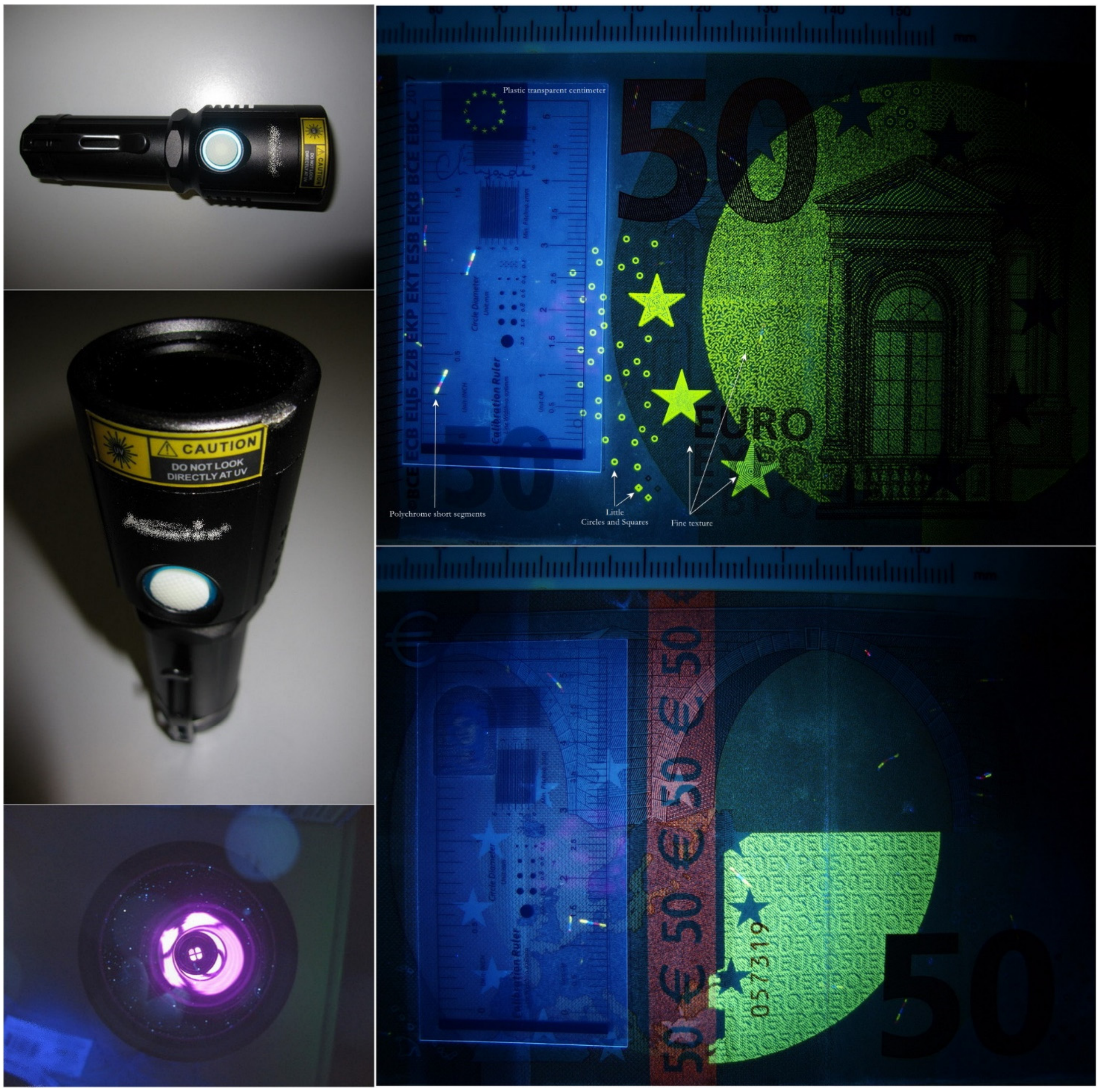

Figure S1. The UVA source.

The test results are listed below:

1. background with a very low blue cast 2. activation of all fluorescent characters extended in a color spectrum including the green, red, magenta, yellow and blue 3. clear

rendering of the texture 4. clear visibility of smaller graphic symbols such as the green circles, squares and the random direction short segments in four colors.

The overlay of a transparent ruler on the left, allows you to estimate the approximate size of the symbols printed on the banknote.

In conclusion, unlike Wood's lights generated by gas tubes, the new devices using UVA LEDs return images with greater brightness and sharpness such as to allow detailed photographs with any digital camera if properly set. It is plausible that the introduction of this technology into clinical routine could bring Wood's light skin examination back to the fore, providing new information even in pathologies not included among those for which the usefulness of UVA was already known. The scabies is an example.
